# The role of SPINK5 mutation distribution in phenotypes of Netherton syndrome

**DOI:** 10.3389/fgene.2025.1475054

**Published:** 2025-01-27

**Authors:** Min Xu, Yujie Shi, Li Lin, Liang Wang, Xianzhong Zhu, Jinglin Xiong, Jiawen Yin, Qing Qi, Wenlin Yang

**Affiliations:** ^1^ Department of Dermatology, The Second Affiliated Hospital of Guangzhou Medical University, Guangzhou, China; ^2^ Department of Neurology, The First Affiliated Hospital, Sun Yat-sen University, Guangzhou, China

**Keywords:** Netherton syndrome, SPINK5, genotype-phenotype correlation, LEKTI, domain

## Abstract

**Objective:**

Netherton syndrome (NS) is a rare hereditary dermatosis, and the correlation between genotype and phenotype in this disease warrants further investigation. This study aimed to explore the genotype-phenotype correlation in NS.

**Methods:**

We collect cases from our clinic and relevant literature. After rigorous screening, we included 162 patients with NS-associated symptoms and SPINK5 mutations. We characterized the distribution and mutation types of allele variants. Logistic regression was employed to analyze the correlation between the location of these variants and phenotypes. Additionally, the association between the homozygous condition of variants and death during infancy was analyzed using the Chi-square test.

**Results:**

Among 162 patients, we identified 324 allele variants, comprising 75 different mutations. Of these, 73 patients carried heterozygous variants, while 89 patients had homozygous variants. We observed that patients with variants or homozygous variants located in the 5′ half of the gene were more likely to experience failure to thrive (*P* < 0.05). Similarly, variants or homozygous variants located outside DomainR-5 were also associated with an increased risk of failure to thrive (*P* < 0.05). Furthermore, variants in domain regions were significantly correlated with the presence of ichthyosis linearis circumflexa (*P* < 0.01). Patients with homozygous fatal variants (c.153delT, c.1431-12G>A, c.1111C>T, c. 1887 + 1G>A, and c. 995delT) had a higher likelihood of mortality during infancy (*P* < 0.001).

**Conclusion:**

Our study provides valuable insights into the genotype-phenotype correlation in Netherton syndrome, enhancing our understanding of the disease and potentially informing the development of future therapeutic approaches.

## 1 Introduction

Netherton syndrome (NS) is a rare autosomal recessive congenital ichthyosis characterized by ichthyosis linearis circumflexa (ILC), erythroderma, hair shaft anomalies, failure to thrive, and recurrent/systematic infections. This disease is caused by mutations in the *SPINK5* gene, which encodes lympho-epithelial Kazal type related inhibitor (LEKTI), a multidomain serine protease inhibitor expressed in the epithelia, mucosa and thymus (Chavanas et al., 2000). LEKTI plays a crucial role in maintaining integrity and protective barrier function of the skin by regulating the activity of defense-activating and desquamation-involved proteases. Mutations in SPINK5 lead to decreased enzyme activity, resulting in epithelial physical barrier defects and the clinical manifestations associated with NS (Jiao et al., 2019). Despite the extensive case reports published since the discovery of SPINK5 pathogenic mutations in NS (Chavanas et al., 2000; Nartisa et al., 2023; Utsumi et al., 2020; Hachem et al., 2006), the genotype-phenotype correlation remains difficult to fully interpret.

The clinical severity of NS can range from mild skin lesions to life-threatening condition, with considerable variability among patients. A previous study suggested a correlation between mutations located upstream of the *SPINK5* gene and more severe phenotypes. However, the lack of a precise definition of the term “upstream” makes it challenging to apply in clinical practice. It is well-established that protein domains serve as functional units of proteins (Wang et al., 2021), and mutations in different domains may have varying impacts on phenotypes (Xu et al., 2018). LEKTI consists of fifteen domains, some of which have been studied in previous biological investigations (Kreutzmann et al., 2004; Wiegmann et al., 2019; Jayakumar et al., 2005; Ramesh et al., 2020). However, the relationship between specific domains and phenotypes in NS has not been thoroughly examined.

In this study, we aimed to investigate the correlation between mutations and clinical phenotypes based on a large cohort collected from previous studies and our clinical practice.

## 2 Methods

### 2.1 Literature search

A systematic search of the Pubmed database was conducted to identify relevant English-language studies on NS using the search terms “Netherton syndrome OR SPINK5 OR LEKTI”. All papers published up to 4 October 2023, were reviewed to determine whether the reported patients met the criteria for inclusion in our study.

### 2.2 Study subjects

Previous studies on NS have limitations, such as small sample sizes or inadequate inclusion criteria (Nartisa et al., 2023; Utsumi et al., 2020; Hachem et al., 2006; Sarri et al., 2017), which may introduce bias into the results. To address these issues, we established stringent inclusion and exclusion criteria for participant selection. After applying these criteria, a total of 73 studies were included, yielding 162 patients: 2 patients from our center (Shi et al., 2017; Xu et al., 2017) and 160 patients reported in the literature. Detailed information on all participants is provided in Supplementary Table S1.

The inclusion criteria were as follows: (1) patients presenting with one or more symptoms of NS; (2) patients with confirmed homozygous or compound heterozygous mutations in the *SPINK5* gene.

The exclusion criteria were as follows: (1) incorrect assessment of gene mutations, such as cDNA changes not conforming to predicted amino acid alterations or reported cDNA base not matching the corresponding base in the reference sequence; (2) presence of two variants of *SPINK5* in a patient where the pathogenic variant could not be determined based on current evidence; (3) identification of only one variant of SPINK5; (4) duplicate records of the same patient across multiple publications; (5) cases involving large deletions or duplications which could not be assigned to certain domains.

### 2.3 Variant analysis

*SPINK5* gene variants were validated using MutationTaster (https://www.mutationtaster.org/) (Schwarz et al., 2014). All mutation data, including cDNA positions and base changes, were standardized according to transcript number NM_006846.4. The transcript was divided into two regions: the 5′ half (1-1598 bp) and 3′ half (1,599–3195 bp). LEKTI consisted of 15 domains, as follows: Domain-1 (AA 28-66), Domain-2 (AA 91-153), Domain-3 (AA 155-216), Domain-4 (AA 219-285), Domain-5 (AA 291-352), Domain-6 (AA 361-423), Domain-7 (AA 431-489), Domain-8 (AA 490-551), Domain −9 (AA 561-622), Domain-10 (AA 626-688), Domain-11 (AA 701 - 757), Domain-12 (AA 768 - 830), Domain-13 (AA 843-905), Domain-14 (AA 910- 971), Domain-15 (AA 987-1,048) (Consortium, 2023). The domains were grouped into five domain regions based on different bio-reactive fragments: DomainR-1 (Domain-1 to Domain-5), DomainR-2 (Domain-6), DomainR-3 (Domain-7), DomainR-4 (Domain-8 to Domain-9), DomainR-5 (Domain-10 to Domain-15) (Sarri et al., 2017). Variants were categorized based on these domains and domain regions for further analysis.

### 2.4 Statistical analysis

This study was designed as a case-control study. Data analysis was performed using SPSS version 24.0 (IBM Corporation, Chicago, IL, United States) and GraphPad PRISM version 7.01 (GraphPad Software, San Diego, CA, United States). The distribution of domain regions and mutation types was described using frequency distributions and pie charts. Binary logistic regression (Enter method) was employed to evaluate the correlation between genotypes (independent variable) and phenotypes (dependent variable), with age included as a covariate due to the age-dependent nature of certain phenotypes. Commonly recorded phenotypes, including ILC, erythroderma, hair shaft anomalies, failure to thrive, recurrent/systematic infections, hypernatremia, angioedema, urticaria, and asthma, were selected for analysis to ensure adequate sample sizes and statistical reliability.

For the assignment of values to independent variables, variants in the 5′ half and 3′ half of the *SPINK5* gene were coded as 1 and 2, respectively. To assess the association between the location of individual domains and phenotypes, variants in different domains were coded according to the domain number. Additionally, variants in DomainR-5 and other domains were assigned values of 1 and 0, respectively. Similarly, variants in domain regions and non-domain regions were assigned values of 1 and 0, respectively. The presence and absence of a phenotype were coded as 1 and 0, respectively. To evaluate the overall effect of two allele variants, we multiplied the values of the two allele variants as independent variables, including Halfx, DomainR-5x, and Domainx.

To investigate genotype-phenotype correlation in patients with homozygous variants, the value of a single allele variant was used, as both alleles were identical. Odds ratios (ORs) with 95% confidence intervals (CIs) were calculated to assess the strength of associations. Contingency tables and Chi-square test were used to investigate the relationship between homozygous condition of fatal mutations and death during infancy. A *P*-value <0.05 was considered statistically significant.

## 3 Results

### 3.1 Basic information of participants

**FIGURE 1:**
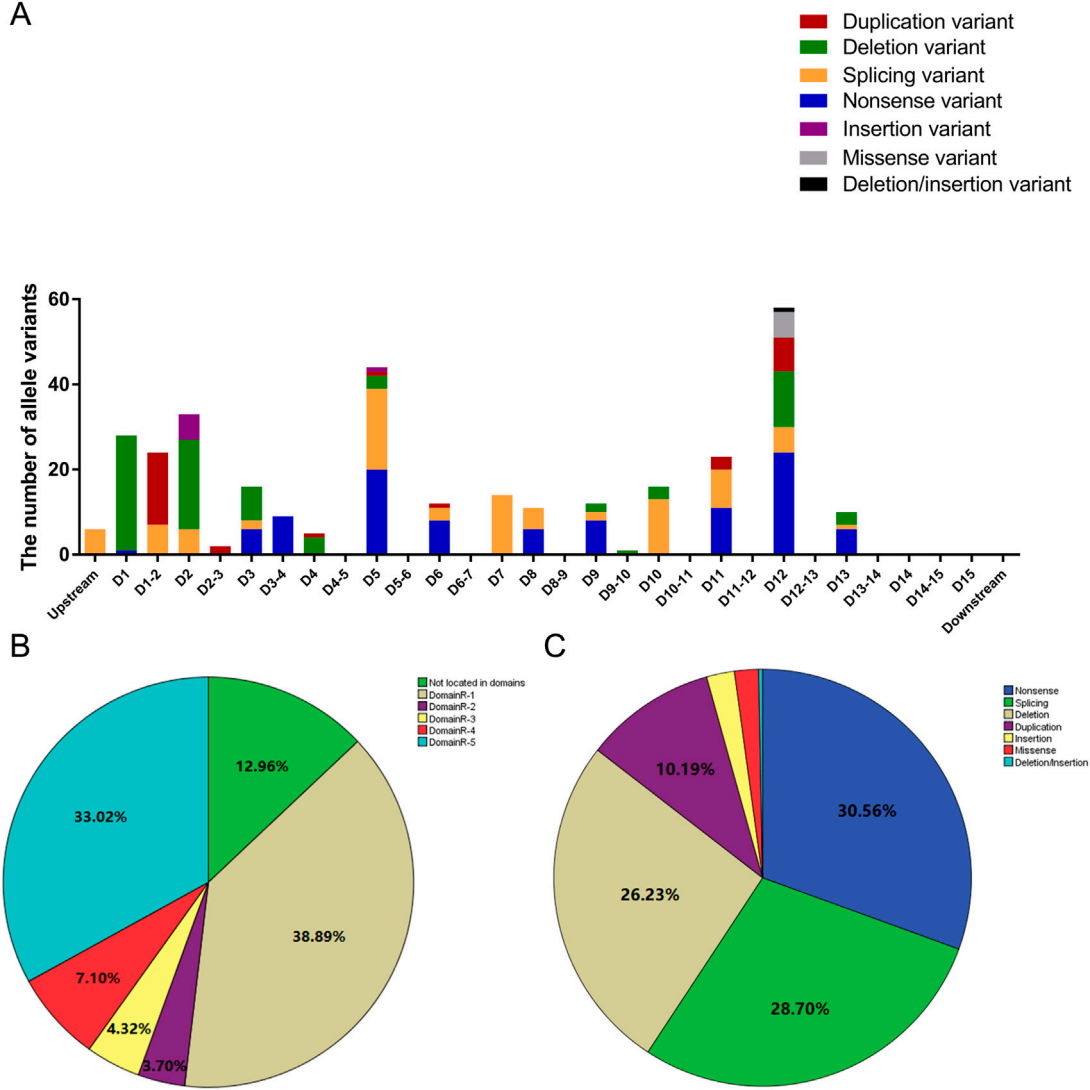
Distribution of allele variants. **(A)** Bar graph illustrating the distribution of allele variants within different domains. D1 represents DomainR-1, D2 represents DomainR-2, D1-2 represents the region between D1 and D2, and so on. **(B)** Percentage of allele variants located in different domain regions. **(C)** Percentage of different mutation types.

A total of 162 participants were included in this study. Among them, 100, 142, 134, 73, 95, 56, 35, 44, and 50 patients had clear clinical records of ILC, erythroderma, hair shaft anomalies, failure to thrive, recurrent/systemic infections, hypernatremia, angioedema, urticaria, and asthma, respectively. Detailed data are presented in Table 1. In total, 324 allele variants and 75 different mutations were identified in the 162 patients. Among them, 73 patients carried heterozygous variants, and 89 carried homozygous variants. The distribution of the 324 allele variants in the *SPINK5* gene is shown in Figure 1A. The majority of variants (87.04%) were located within domain regions, with the 5′-terminus (DomainR-1) and 3′-terminus (DomainR-5) representing mutation hotspots (Figure 1B). The most common mutation types observed in patients were nonsense, splicing, and deletion mutation (Figure 1C). Detailed genotype information is provided in Table 2.

**TABLE 1:** Information of phenotypes of participants.

Clinical phenotypes	N (%)		
	+	−	NA
ILC	73 (45.06)	27 (16.67)	62 (38.27)
Erythroderma	124 (76.54)	18 (11.11)	20 (12.35)
Hair shaft anomalies	120 (74.07)	14 (8.64)	28 (17.28)
Failure to thrive	56 (34.57)	17 (10.49)	89 (54.94)
Recurrent/systemic infection	79 (48.76)	16 (9.88)	67 (41.36)
Hypernatremia	31 (19.14)	25 (15.43)	106 (65.43)
Angioedema	12 (7.41)	23 (14.20)	127 (78.39)
Urticaria	12 (7.41)	32 (19.75)	118 (72.84)
Asthma	16 (9.88)	34 (20.99)	112 (69.13)

Abbreviations: ILC, ichthyosis linearis circumflexa; NA, not available.

**TABLE 2:** Information of genotype in participants.

Allele variants	N (%)
Variant type	
Nonsense	99 (30.56)
Splicing	93 (28.70)
Deletion	85 (26.23)
Duplication	33 (10.19)
Insertion	7 (2.16)
Missense	6 (1.85)
Deletion/insertion	1 (0.31)
Domain region	
DomainR-1	126 (38.89)
DomainR-2	12 (3.70)
DomainR-3	14 (4.32)
DomainR-4	23 (7.10)
DomainR-5	107 (33.02)
Not located in domains	42 (12.96)

### 3.2 The location of allele variants was correlated with clinical phenotypes

**FIGURE 2:**
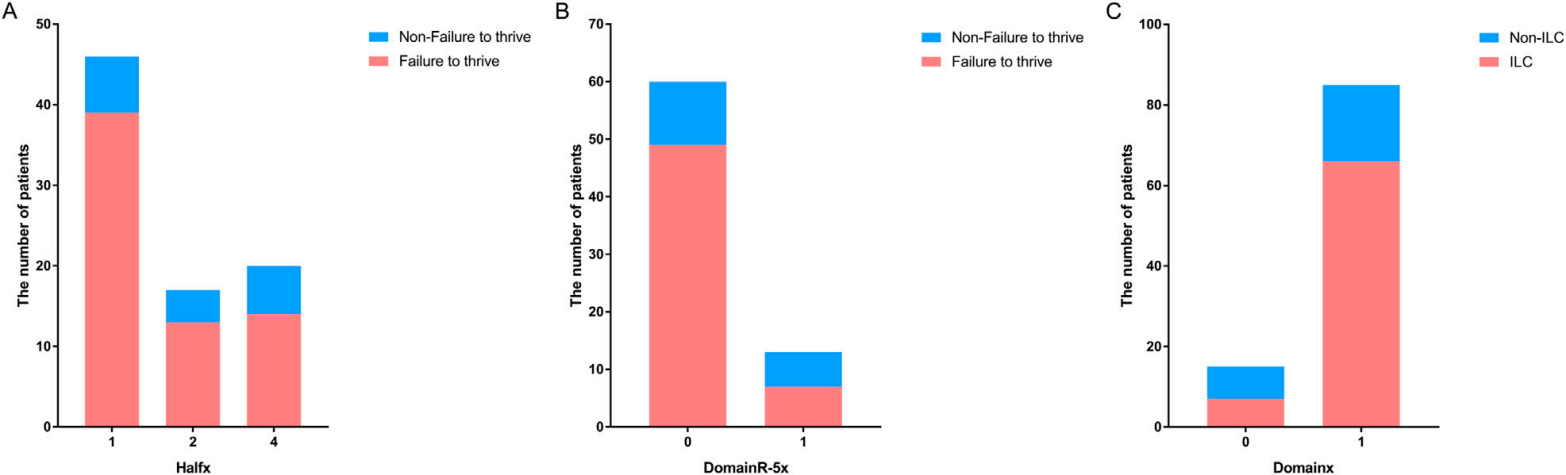
Distribution of patients with variants in different locations of SPINK5. Bar graphs showing **(A)** the number of patients with variants located upstream and downstream in the non-failure to thrive group *versus* the failure to thrive group, **(B)** the number of patients with variants located in DomainR-5 and other domains in the non-failure to thrive group *versus* the failure to thrive group. **(C)** The number of patients with variants located in domains and non-domain regions in the non-ILC group *versus* the ILC group.

A previous study suggested that variants located upstream of the *SPINK5* gene are associated with more severe phenotypes (Sarri et al., 2017). To further investigate this, we defined 5′ half as upstream and 3′ half as downstream. Our analysis confirmed this trend, showing that patients with variants located in the 5′ half of the gene tended to experience failure to thrive [*P* < 0.05, OR (95% CI): 0.605 (0.384–0.954); Figure 2A]. Furthermore, a detailed analysis of the correlation between phenotypes and variants in different domains revealed that patients with variants in upstream domains were more likely to experience failure to thrive [*P* < 0.05, OR (95%): 0.987 (0.977–0.988)]. When analyzing domain regions, variants outside of DomainR-5 were more strongly associated with failure to thrive [*P* < 0.05, OR (95%): 0.250 (0.066–0.942); Figure 2B], suggesting that mutations in specific domain regions have a significant impact on clinical phenotypes. Additionally, patients with variants in domains were more likely to have ILC [*P* < 0.01, OR (95%): 6.385 (1.722–23.679); Figure 2C]. No significant correlations were found between individual domains and other phenotypes; detailed information is provided in Supplementary Table S2.

### 3.3 The location of homozygous variants was correlated with clinical phenotypes

**FIGURE 3:**
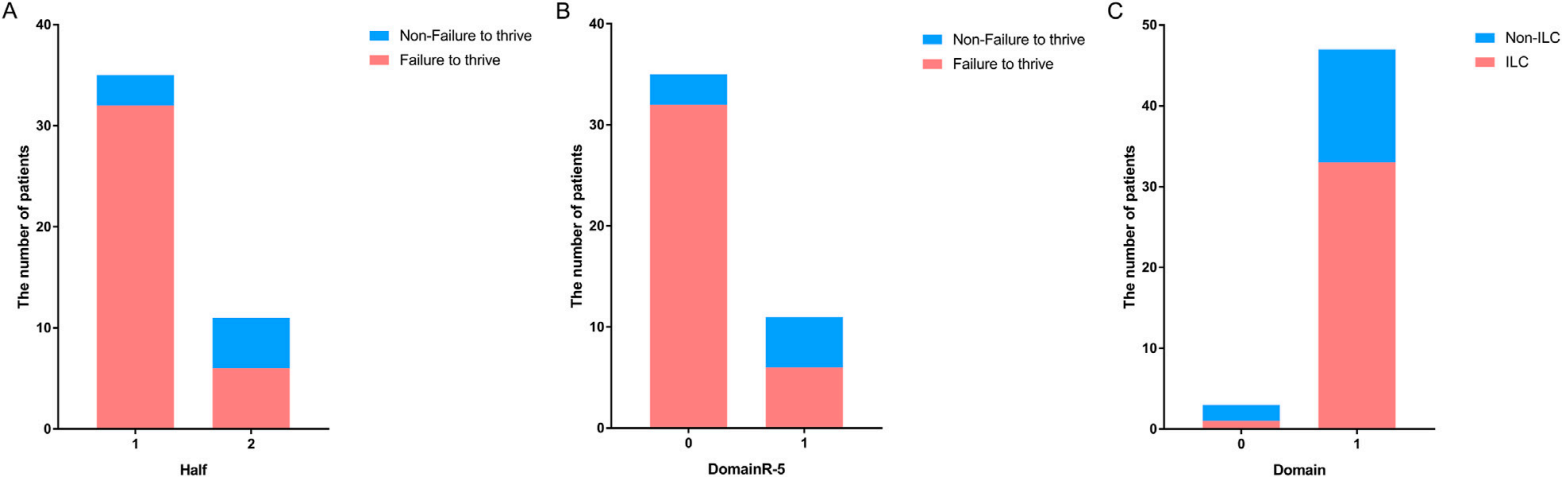
Distribution of patients with homozygous variants in different locations of SPINK5. Bar graphs showing **(A)** the number of patients with homozygous variants located upstream and downstream in the non-failure to thrive group *versus* the failure to thrive group, **(B)** the number of patients with homozygous variants located in DomainR-5 and other domains in the non-failure to thrive group *versus* the failure to thrive group. **(C)** The number of patients with homozygous variants located in domains and non-domain regions in the non-ILC group *versus* the ILC group.

To further validate these findings, we performed the same analysis in patients with homozygous variants. The results revealed that patients with homozygous variants located in the 5′ half of the gene [*P* < 0.05, OR (95% CI): 0.121 (0.020–0.720); Figure 3A] or upstream domains [*P* < 0.05, OR (95%): 0.769 (0.618–0.956)] or outside of DomainR-5 [*P* < 0.05, OR (95%): 0.121 (0.020–0.720); Figure 3B] were more likely to experience failure to thrive. However, although a tendency was observed, no significant correlation was found between ILC and whether the variants were located in domains [*P* > 0.05, OR (95% CI): 8.339 (0.573–131.243); Figure 3C]. The lack of correlation may be attributed to the small number of patients with variants outside domains (N = 3). No significant correlations were found between individual domains and other phenotypes; detailed information is provided in Supplementary Table S3.

### 3.4 The genotype-phenotype correlation in fatal variants

The most severe phenotype observed in this study was death during infancy. Fourteen patients died during infancy, all of whom carried homozygous fatal variants, including c.153delT, c.1431-12G>A, c.1111C>T, c. 1887 + 1G>A, and c. 995delT. Notably, most of these fatal variants were located at the 5′ terminus of DomainR-5.

Furthermore, 18 patients with homozygous fatal variants and 12 patients with heterozygous fatal variants were identified. None of the patients with heterozygous fatal variants died during infancy, and this difference was statistically significant (*P* < 0.001). These findings suggest that homozygous fatal variants are strongly associated with premature death. Detailed data are presented in Table 3.

**TABLE 3:** Genotypes of patients died during infancy.

Variants	Half	Domain	DomainR	No. of patients with homozygous variants (death/total)	No. of patients with heterozygous variants (death/total)
c.153delT	1	1	1	8/11^a^	c.81 + 2 T>A (0/2) c.891C>T (0/3)^a^
c.1431-12G>A	1	7	3	3/3	c.1816_1820 + 21delinsCT (0/1)^a^ c.891C>T (0/1) c.2472_2473delAG (0/1)
c.1111C>T	1	6	2	1/1	c.2468delA (0/1) c.1032_1036dupGAAAA (0/1) c.81 + 2 T>A (0/1) c.2041_2042delAG (0/1)
c.1887 + 1G>A	2	10	5	1/2	—
c.995delT	1	5	1	1/1	—

^a^ One of the live patients was less than 1 year old.

## 4 Discussion

The phenotypic spectrum of Netherton syndrome ranges from mild skin lesions to a potentially fatal condition. However, the genotype-phenotype correlation in this syndrome remains poorly understood. In our study, we focused on recording the clearly described phenotypes from the literature, ensuring the accuracy of the results. Our findings revealed that domainR-1 and domainR-5 were mutation hotspots. The phenotypes of ILC, failure to thrive, and death during infancy were correlated with the genotype.

A previous study reported that mutations located upstream in the *SPINK5* gene are associated with more severe phenotypes (Sarri et al., 2017), and our findings corroborate this observation. Furthermore, we employed a more precise localization approach, analyzing specific domains, and found that the phenotypes were correlated with the specific domain regions where the variants were located. Our results suggest that DomainR-5 may not play a critical role in maintaining the function of *SPINK5* compared to other domain regions, which is consistent with previous biological studies (Kreutzmann et al., 2004; Wiegmann et al., 2019; Jayakumar et al., 2005; Ramesh et al., 2020). Notably, most fatal variants associated with death during infancy were also located upstream of domainR-5. The only fatal variant within domainR-5, c.1887 + 1G>A, is closed to domainR-4, further supporting the hypothesis that mutations in domainR-5 are probably associated with milder phenotypes.

Five fatal variants were identified in our study, all of which resulted in death during infancy. Significantly, the high mortality rate was observed exclusively in the homozygous condition. When these fatal variants were present in a heterozygous condition, in combination with a non-fatal variant, the patients were able to survive. This observation may be attributed to the loss of function pathogenesis of *SPINK5* (Chavanas et al., 2000). In the presence of a non-fatal variant, which may retain partial enzyme function, the negative effects of the fatal variant are partially alleviated. This finding suggests that gene editing technology could be beneficial in treating this disease, even if it may introduce new mutations into the other allele. Additionally, it is noteworthy that three patients with homozygous fatal variants did not die during infancy. Two of them carried the c.153delT variant, and one carried the c.1887 + 1G>A variant. This suggests that other factors such as modifier genes and epigenetic influences, may influence the phenotypes and warrants further investigation.

There are some limitations in this study. First, although we considered age as a cofactor to reduce bias, certain phenotypes may evolve or resolve as patients grow older. Thus, our results would benefit from validation in a longitudinal cohort. Second, to ensure data accuracy, we excluded certain types of patients, such as those with large deletions or duplications of the gene. Third, variability in medical condition and disease management across different geographic locations may affect disease phenotypes, such as growth and infection status, which could limit the generalizability of our findings.

In conclusion, our study provides valuable insights into the genotype-phenotype correlation in Netherton syndrome, specially highlighting the correlation between domain regions and phenotypes. This enhances our understanding of the disease and potentially guides the development of future therapeutic approaches and improved disease management.

## Data Availability

The datasets presented in this study can be found in online repositories. The names of the repository/repositories and accession number(s) can be found in the article/Supplementary Material.
